# Trends and projections of overweight and obesity among Chinese college students from 1995 to 2019: Findings from national cross-sectional surveys

**DOI:** 10.1371/journal.pone.0337477

**Published:** 2026-01-07

**Authors:** Pengying Du, Jianhua Zhang, Ling Zhang

**Affiliations:** 1 School of Physical Education and Health, Ningxia Medical University, Yinchuan, China; 2 School of Physical Education and Art, Hunan University of Medicine, Huaihua, China; 3 School of Sports Science, Jishou University, Jishou, China; 4 School of Public Health, Ningxia Medical University, Yinchuan, China; University of Potsdam, GERMANY

## Abstract

**Background:**

This paper aimed to investigate the prevalence of overweight and obesity among Chinese college students aged 19–22 years from 1995 to 2019 and projections to 2030.

**Methods:**

Data among 291,276 Han Chinese college students aged 19–22 years were obtained from the Chinese National Surveillance on Students’ Constitution and Health from 1995 to 2019. Weight categories were based on standardized weight-for-height values by sex and age. The chi-square trend test was used to estimate trends in prevalence across subgroups. Log-binomial regression was used to estimate the prevalence odds ratios.

**Results:**

The prevalence of overweight and obesity among college students aged 19–22 years progressively increased from 3.7% and 0.7% in 1995 to 23.4% and 11.4%, respectively, in 2019 (*P* < 0.001). Significant increasing trends were observed in all the subgroups (*P* < 0.001). The increases in the prevalence of overweight and obesity accelerated. The risk of overweight (prevalence odds ratios (PORs): 1.42 ~ 3.01, *P* < 0.001) and obesity (PORs: 2.82 ~ 4.42, *P* < 0.001) was greater for males from 1995 to 2019 and for urban students (overweight: 1.21 ~ 1.63, *P* < 0.001; obesity: 1.31 ~ 1.97, *P* = 0.004~<0.001) from 2000 to 2019. The risk was lower for urban students in 1995 than for rural students. In recent years, older college students have been at greater risk. Based on our findings, we expect that the prevalence of overweight and obesity in all subgroups will continue to increase from 2025 to 2030. The prevalence of overweight among rural college students can be predicted to exceed that among urban college students after 2027.

**Conclusion:**

The prevalence of overweight and obesity among Chinese college students increased from 1995 to 2019. Future policies should focus on high-risk groups, especially rural and male students, to improve the health and equity of college students.

## Introduction

The prevalence of overweight and obesity has become a serious public health problem worldwide, and the prevalence of overweight and obesity continues to rise globally, including in China [1–3]. From 1990 to 2022, the global prevalence of obesity among children and adolescents aged 5–19 years increased from 2% to 8%, and that among adults aged 18 years and older increased from 7% to 16% [1,4]. In 2014, China ranked first among 200 countries in the number of males and females with obesity, surpassing that of the United States [5]. Being overweight or obese is associated with health risks as well as premature death from cardiovascular and respiratory diseases, diabetes, musculoskeletal disorders, infertility, and some cancers [4,6].

Most college students are in early adulthood (18–25 years), which is an important transition period for individuals going from adolescence to adulthood, and the development of a healthy lifestyle during this period is crucial for reducing or preventing the incidence of chronic noncommunicable diseases in adulthood [7]. The total number of junior college students and undergraduate students in China has increased from 1.7 million in 1985 to 37.75 million in 2022 [8]. College students are the main force in the future construction of the country, so it is necessary to determine the health status of such a large population. Several studies have investigated cross-sectional results on the prevalence of overweight and obesity among college students in countries with different incomes [7,9]. with evidence from 50 universities in China reporting a prevalence of 9.5% [10]. The prevalence of overweight and obesity among college students in Norway and Cameroon has increased from 23.5% and 3.0% to 24.8% and 4.9%, respectively, in the last decade [11,12]. The same upward trends were reported for Thai royal army conscripts aged 18–24 years and Swiss male conscripts aged 18–19 years [13,14]. There has also been much evidence from young citizens in various countries since the 21st century, reporting that the prevalence of overweight and obesity has increased in Korea [15,16], Spain [17], Brazil [18], France [19], and Ghana [20], but not in Saudi Arabia [21], where the prevalence has declined. However, there is little evidence in China. Many efforts have been made in China to investigate the prevalence of overweight and obesity among children and adolescents as well as adults [2,3]. However, few investigations have been conducted on populations during the transition period, and the prevalence among Chinese college students has remained unknown over the past few decades.

As the world's largest developing country, the changes observed in China may correspond to or predict demographic changes in other Asian countries and low- and middle-income countries, and the results of this study may provide valuable insights for other low- and middle-income countries. Therefore, by the Chinese National Surveillance on Students’ Constitution and Health (CNSSCH) from 1995 to 2019, this paper aimed to investigate temporal trends in the prevalence of overweight and obesity over the past three decades and projections in prevalence for 2025 and 2030.

## Materials and methods

### Study design and subjects

Data from Han Chinese college students aged 19–22 years were obtained from published summary statistics by the CNSSCHs in 1995, 2000, 2005, 2010, 2014 and 2019 [22–27]. The CNSSCH used a multistage stratified cluster sampling design. The study included only Han Chinese college students. The Han population made up 91% of the total Chinese population. Thirty provinces/municipalities/autonomous regions, with the exceptions of Tibet, Hong Kong, Macau and Taiwan, participated in the survey. Since 1985, college students aged 19–22 years from three universities in each province who were representative of the physical fitness and health status of college students from their original province were selected. Students from their original province are those who have graduated from high school and entered university in their province. Then, participants were stratified by urban or rural area according to their place of residence. Participants were excluded from the study based on the following criteria: (1) diagnosis of major organ diseases affecting cardiac, pulmonary, hepatic, or renal systems; (2) presence of clinically significant growth disorders, including but not limited to dwarfism or gigantism; (3) physical disabilities or congenital deformities impacting functional mobility; (4) active acute illness at enrollment or history of acute illness during the final month of the study period without full recovery; and (5) current menstruation status in female participants during testing procedures. Since 1985, the sample of schools has remained almost the same for each survey. From 1995 to 2019, data were obtained for 145,938 males and 145,338 females. The sex-, region- and age-specific sample sizes among Chinese college students in each survey year are shown in Table 1. Verbal informed consent was obtained from each participant and their parents.

**Table 1:** Sample sizes of Chinese college students aged 19-21 years from 1985 to 2019.

	Males						Females					
	1995	2000	2005	2010	2014	2019	1995	2000	2005	2010	2014	2019
Urban												
19	2714	3401	3766	2962	2968	2774	2882	3547	3937	2996	2996	2836
20	2762	3279	3737	2967	2963	2866	2892	3406	3904	2982	2988	2863
21	2730	3287	3728	2968	2984	2771	2883	3057	3752	2984	2987	2783
22	2691	2818	3318	2912	2856	2548	2738	2655	3390	2917	2881	2529
19-22	10897	12785	14549	11809	11771	10959	11395	12665	14983	11879	11852	11011
Rural												
19	2760	3366	3654	2993	2978	2683	2652	3252	3696	2978	2996	2754
20	2805	3391	3627	2997	2981	2788	2782	3149	3546	2992	2995	2857
21	2831	3554	3470	2992	2983	2785	2764	3181	3299	2992	3000	2833
22	2848	3143	3182	2941	2978	2438	2635	2678	3120	2945	2986	2471
19-22	11244	13454	13933	11923	11920	10694	10833	12260	13661	11907	11977	10915
Total												
19	5474	6767	7420	5955	5946	5457	5534	6799	7633	5974	5992	5590
20	5567	6670	7364	5964	5944	5654	5674	6555	7450	5974	5983	5720
21	5561	6841	7198	5960	5967	5556	5647	6238	7051	5976	5987	5616
22	5539	5961	6500	5853	5834	4986	5373	5333	6510	5862	5867	5000
19-22	22141	26239	28482	23732	23691	21653	22228	24925	28644	23786	23829	21926

### Measurements

The test time for these surveys ranged from September to November. All the measuring instruments used were consistent in each survey year and were calibrated before use. All participants were given complete tests at all survey sites following the same protocol. The staff at all survey sites received rigorous training. The apparatus recommended by Cameron [28] was used by doctors to measure height and weight. Height was measured to the nearest 0.1 cm by a stadiometer. The participant stood barefoot, with his/her back to the column, on the base plate of the stadiometer. The trunk was naturally straight, the head was straight, and the eyes were looking straight ahead. The upper limbs drooped naturally, the legs were straight, and the heels of the feet were together with the toes separated by approximately 60°. The heel, sacrum, and scapula contacted the column in the “three points and one line” standing position. The staff slid the horizontal pressure plate down the column to the top of the participant's head and then read. Body weight was measured to the nearest 0.1 kg by an electronic scale, with all participants wearing underwear and barefoot. The working conditions, accuracy, and sensitivity of the electronic weighing scale were examined. The participant, wearing underwear and barefoot, stood naturally in the center of the measurement plate and kept his/her body steady. After the value shown on the display stabilized, the staff recorded the displayed value. Weight categories among college students aged 19–22 years were based on standardized weight-for-height criteria from the 80th percentile weight to the same height population by sex and age. These criteria were based on the 1985 CNSSCH data. Overweight was defined as an individual's weight being 110% or more of the standard weight for height, and obesity was defined as weight being 120% or more [22]. Overweight always includes obesity.

### Statistical analysis

The prevalence of overweight and obesity was extracted from published summary statistics for different survey years according to sex, region, and age. The chi-square trend test was used to estimate trends in prevalence across subgroups. The data were divided into three periods: 10 years of data from 1995 to 2005, 9 years of data from 2005 to 2014, and 5 years of data from 2014 to 2019, and the annual change was calculated for each period separately. The annual changes for the three periods were compared to assess changes in the trend in prevalence from 1995 to 2019. To assess the factors influencing prevalence at each time point, log-binomial regression was used to estimate the prevalence odds ratios (PORs) and 95% confidence intervals (CIs), adjusting for sex (male, female), region (urban, rural), and age (19–20 years, 21–22 years) in the model. Polynomial regression models were used to analyze trends in each detection rate from 1995 to 2019, with the year as the independent variable and the detection rates as the dependent variables. Models were built separately for sex, region, and age categories. These models were used to predict detection rates for different subgroups among college students in 2025 and 2030. All the statistical analyses were performed by SPSS 27.0 and GraphPad Prism 9.3.1. Two-sided P values < 0.05 were considered significant.

## Results

### Trends in the prevalence of overweight and obesity

For the total population, the prevalence of overweight and obesity among college students aged 19–22 years progressively increased from 3.7% and 0.7% in 1995 to 23.4% and 11.4%, respectively, in 2019 (*P* < 0.001). Significant increasing trends were observed in all subgroups (*P* < 0.001). The annual changes in the prevalence of overweight and obesity progressively increased during three periods—from 1995 to 2005, 2005–2014, and 2014–2019. The annual changes in all detection rates were greater for urban and older students than for rural and younger students in all three periods. Stratifying by sex, the prevalence of overweight and obesity among males increased from 3.1% and 0.8% to 36.6% and 16.9%, respectively; for females, they increased from 4.3% and 0.5% to 13.9% and 5.9%, respectively (*P* < 0.001). The annual changes from 2014 to 2019 were the greatest of the three periods. The temporal trends were consistent with those of the total sample in all subgroups. The annual changes for urban males and females were greater than those for rural males and females in most periods, but the annual changes for urban were smaller than those for their rural counterparts among males from 2014 to 2019 and females from 2005 to 2014 (Table 2). The prevalence of overweight and obesity among Chinese college students aged 19–22 years by sex, region, and age from 1995 to 2019 is shown in S1 Table.

**Table 2:** Prevalence of overweight and obesity by survey year, sex, age and region [n (%)].

Survey year	Overweight					Obesity				
	Total	Urban	Rural	19-20 years	21-22 years	Total	Urban	Rural	19-20 years	21-22 years
Total										
1995	1648 (3.7)	699 (3.1)	949 (4.3)	835 (3.8)	813 (3.7)	307 (0.7)	165 (0.7)	142 (0.6)	168 (0.8)	139 (0.6)
2000	4601 (9.0)	2771 (10.9)	1830 (7.1)	2333 (8.7)	2268 (9.3)	1330 (2.6)	867 (3.4)	463 (1.8)	674 (2.5)	656 (2.7)
2005	6036 (10.6)	3750 (12.7)	2286 (8.3)	3104 (10.4)	2932 (10.8)	1969 (3.4)	1330 (4.5)	639 (2.3)	1020 (3.4)	949 (3.5)
2010	6251 (13.2)	3588 (15.1)	2663 (11.2)	3071 (12.9)	3180 (13.4)	2190 (4.6)	1331 (5.6)	859 (3.6)	1117 (4.7)	1073 (4.5)
2014	8090 (17.0)	4571 (19.3)	3519 (14.7)	3904 (16.4)	4186 (17.7)	3055 (6.4)	1823 (7.7)	1232 (5.2)	1486 (6.2)	1569 (6.6)
2019	10212 (23.4)	5631 (25.6)	4581 (21.2)	5100 (22.7)	5112 (24.2)	4955 (11.4)	2833 (12.9)	2122 (9.8)	2458 (11.0)	2497 (11.8)
*χ*^2^ trend value	9469.952	5366.340	4273.333	4564.985	4911.134	6885.851	3644.744	3338.545	3292.121	3601.734
*P* value	<0.001	<0.001	<0.001	<0.001	<0.001	<0.001	<0.001	<0.001	<0.001	<0.001
Annual change from 1995 to 2005	0.69	0.96	0.40	0.66	0.71	0.28	0.38	0.17	0.26	0.29
Annual change from 2005 to 2014	0.72	0.74	0.72	0.67	0.77	0.33	0.36	0.32	0.31	0.34
Annual change from 2014 to 2019	1.28	1.26	1.29	1.26	1.30	0.99	1.04	0.93	0.96	1.04
Males										
1995	697 (3.1)	223 (2.0)	474 (4.2)	324 (2.9)	373 (3.4)	187 (0.8)	96 (0.9)	91 (0.8)	100 (0.9)	87 (0.8)
2000	3101 (11.8)	1980 (15.5)	1121 (8.3)	1519 (11.3)	1582 (12.4)	991 (3.8)	664 (5.2)	327 (2.4)	487 (3.6)	504 (3.9)
2005	4240 (14.9)	2713 (18.6)	1527 (11.0)	2077 (14.0)	2163 (15.8)	1525 (5.4)	1047 (7.2)	478 (3.4)	759 (5.1)	766 (5.6)
2010	4688 (19.8)	2742 (23.2)	1946 (16.3)	2243 (18.8)	2445 (20.7)	1785 (7.5)	1094 (9.3)	691 (5.8)	898 (7.5)	887 (7.5)
2014	5906 (24.9)	3408 (29.0)	2498 (21.0)	2756 (23.2)	3150 (26.7)	2383 (10.1)	1430 (12.1)	953 (8.0)	1136 (9.6)	1247 (10.6)
2019	7178 (33.2)	4016 (36.6)	3162 (29.6)	3508 (31.6)	3670 (34.8)	3659 (16.9)	2117 (19.3)	1542 (14.4)	1774 (16.0)	1885 (17.9)
*χ*^2^ trend value	8468.813	4786.027	3866.789	4013.578	4469.165	5274.961	2765.802	2580.546	2457.319	2828.142
*P* value	<0.001	<0.001	<0.001	<0.001	<0.001	<0.001	<0.001	<0.001	<0.001	<0.001
Annual change from 1995 to 2005	1.17	1.66	0.67	1.11	1.24	0.45	0.63	0.26	0.42	0.48
Annual change from 2005 to 2014	1.12	1.15	1.11	1.02	1.21	0.52	0.55	0.51	0.50	0.56
Annual change from 2014 to 2019	1.64	1.54	1.72	1.68	1.62	1.37	1.43	1.28	1.28	1.46
Females										
1995	951 (4.3)	476 (4.2)	475 (4.4)	511 (4.6)	440 (4.0)	120 (0.5)	69 (0.6)	51 (0.5)	68 (0.6)	52 (0.5)
2000	1500 (6.0)	791 (6.2)	709 (5.8)	814 (6.1)	686 (5.9)	339 (1.4)	203 (1.6)	136 (1.1)	187 (1.4)	152 (1.3)
2005	1796 (6.3)	1037 (6.9)	759 (5.6)	1027 (6.8)	769 (5.7)	444 (1.6)	283 (1.9)	161 (1.2)	261 (1.7)	183 (1.3)
2010	1563 (6.6)	846 (7.1)	717 (6.0)	828 (6.9)	735 (6.2)	405 (1.7)	237 (2.0)	168 (1.4)	219 (1.8)	186 (1.6)
2014	2184 (9.2)	1163 (9.8)	1021 (8.5)	1148 (9.6)	1036 (8.7)	672 (2.8)	393 (3.3)	279 (2.3)	350 (2.9)	322 (2.7)
2019	3034 (13.8)	1615 (14.7)	1419 (13.0)	1592 (14.1)	1442 (13.6)	1296 (5.9)	716 (6.5)	580 (5.3)	684 (6.0)	612 (5.8)
*χ*^2^ trend value	1846.637	1029.153	833.606	910.890	945.720	1849.480	970.424	897.373	926.224	928.314
*P* value	<0.001	<0.001	<0.001	<0.001	<0.001	<0.001	<0.001	<0.001	<0.001	<0.001
Annual change from 1995 to 2005	0.20	0.27	0.12	0.22	0.17	0.10	0.13	0.07	0.11	0.08
Annual change from 2005 to 2014	0.32	0.32	0.33	0.31	0.33	0.14	0.16	0.13	0.13	0.16
Annual change from 2014 to 2019	0.93	0.97	0.90	0.90	0.98	0.62	0.64	0.60	0.62	0.62

### Influence of sex, region and age on prevalence by survey year

**Fig 1:**
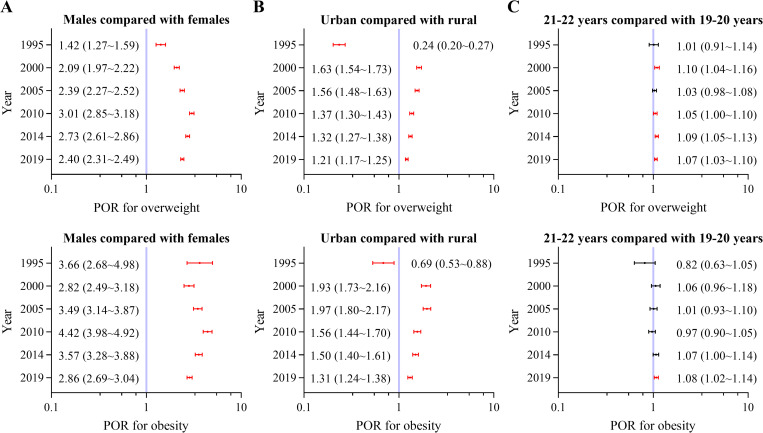
Prevalence odds ratios (PORs) with 95% confidence intervals (CIs) for overweight and obesity across years. (A) female college students aged 19-22 years compared with male college students aged 19-22 years adjusted for age and region based on males as a reference; (B) rural college students aged 19-22 years compared with urban college students aged 19-22 years adjusted for sex and age based on urban college students as a reference; (C) college students aged 19-20 years compared with college students aged 21-22 years adjusted for sex and region based on college students aged 21-22 years as a reference. Red represents *P* < 0.05, and black represents *P* > 0.05.

The risks of overweight and obesity were consistently greater in males than in females (*P* < 0.001). The sex PORs for overweight consistently increased from 1.42 in 1985 to 3.01 in 2010 and then consistently decreased to 2.40 in 2019. The sex PORs for obesity fluctuated slightly across the years (Fig 1A). The prevalence of overweight and obesity was greater among rural college students than those of among urban college students in 1995, but thereafter the prevalence was greater among urban college students (*P* < 0.05). The regional PORs increased slightly from 2000 to 2019 (*P* < 0.05) (Fig 1B). For college students aged 19–20 years, the risk of obesity in 2019, and the risk of overweight in 2000 and from 2010 to 2019 were lower than those for college students aged 21–22 years (Fig 1C).

### Projection of overweight and obesity

**Fig 2:**
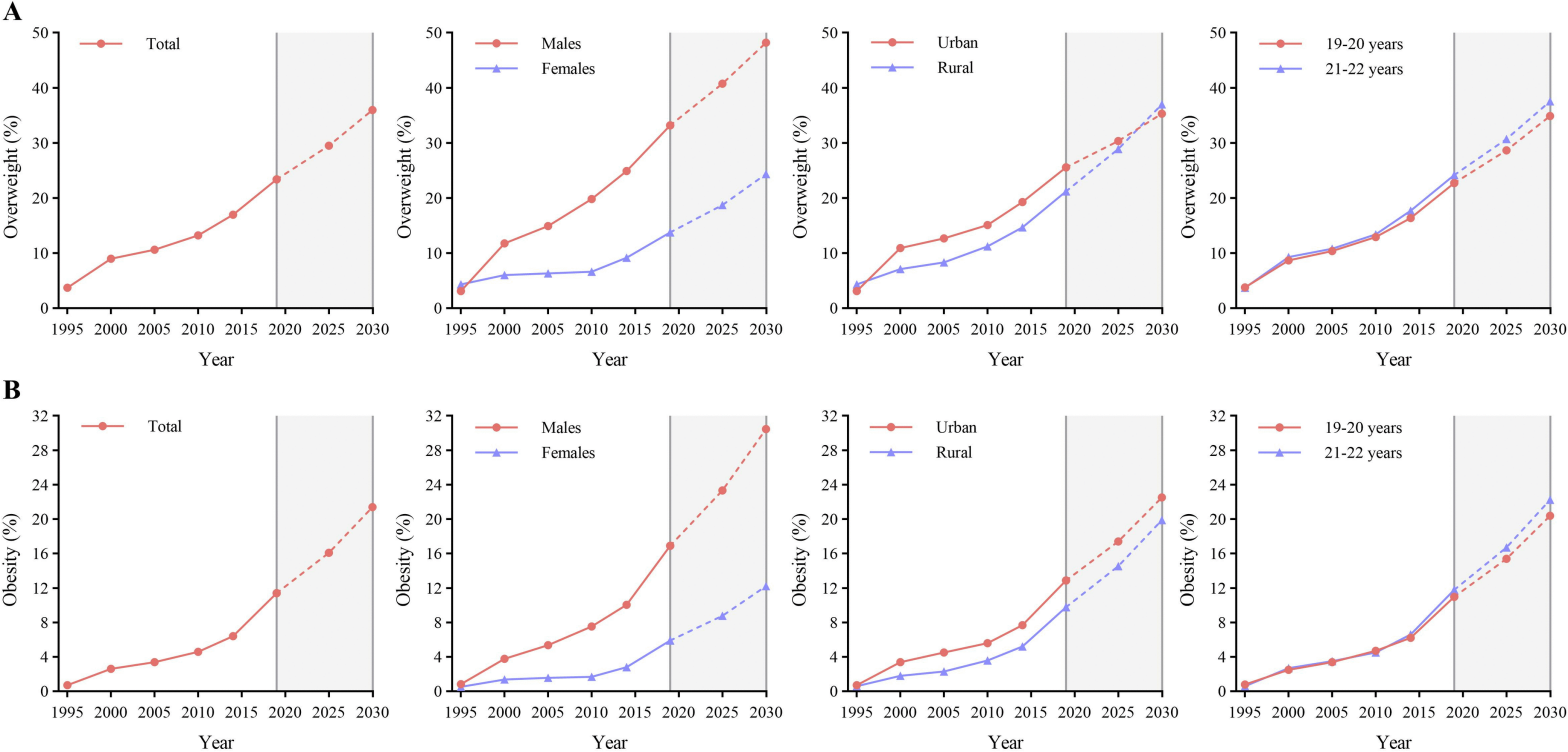
Prevalence of overweight and overweight from 1995 to 2019 and projections to 2030 by sex, region and age. **(A): overweight; (B): obesity.** Overweight was defined as an individual's weight being 110% or more of the standard weight for height, and obesity was defined as weight being 120% or more. Overweight always includes obesity.

Using polynomial regression prediction models, the prevalence of overweight and obesity was projected to continue to increase in all subgroups among college students. The prevalence of overweight and obesity among the total population is projected to reach 36.0% and 21.4%, respectively, by 2030. The sex difference in the prevalence of overweight increased from 19.4% in 2019 to 23.8% in 2030. The prevalence of overweight among urban and rural college students will reach 35.3% and 37.0%, respectively, in 2030, with the prevalence among rural college students exceeding that among urban college students after 2027. The prevalence of overweight and obesity were greater among college students aged 19–20 years than among college students aged 21–22 years from 2019 to 2030. The difference across age categories in the prevalence of overweight and obesity increased from 2019 to 2030 (Fig 2).

## Discussion

This paper provided the first estimate of trends in the prevalence of overweight and obesity among college students in China from 1995 to 2019. The prevalence of overweight and obesity has continued to increase over the past two decades, and the trend has accelerated. In general, males and urban college students had greater risk of overweight or obesity.. It was projected that by 2030, there will be more than one-third of college students with overweight or obesity, and the prevalence of overweight among rural college students would exceed that among urban college students. Notably, the prevalence of overweight and obesity in recent years was also greater among older college students. Future interventions should prioritize high-risk groups to curb the prevalence of overweight and obesity.

The steadily increasing trend in the prevalence of overweight and obesity among college students in China over time was consistent with those reported for populations in similar age groups in other countries [11,12,14,16–18,20]. Interestingly, the prevalence of obesity among males and females aged 18–29 years in France increased from 3.5% and 5.6% to 6.8% and 8.3%, respectively, from 2013–2016, but no significant trend was observed among adults older than 29 years [19]; the prevalence of obesity among Royal Thai Army conscripts aged 18–24 years increased from 23.7% to 28.4% from 2017 to 2021, and there was little or no significant increase among older populations [13]. The prevalence of overweight among youth aged 18–25 years in Saudi Arabia decreased from 41% to 24% from 2012 to 2021 [21]. Unfortunately, there are few findings on trends for Chinese college students or groups in this age group. The results for adults aged 18–39 years were reported in a survey of Chinese adults, with the prevalence of overweight increasing from 24.0% in 1994 to 46.7% in 2015 [3]. Notably, the interpretation of trends must take into account changes in population structure. According to China's national census data, the proportion of college students among the population aged 19–22 years increased rapidly from 4.1% in 2000 to 28.1% in 2020, reflecting the expanding scale of higher education and making college students an important group among the youth population [29]. In earlier years, college students were more likely to come from urban areas and families with higher socioeconomic status (SES) (who could afford education costs) [30]. However, over time, economic development and national policy inclination have led to a sharp decline in China's poverty population and percentage [31], with more families able to afford their children's education expenses. Additionally, to ensure equitable access to education, China has continued to advance special plans for the enrollment and financial assistance of college students from impoverished areas. According to data from the Ministry of Education, the number of enrolled college students under these special plans increased from 10,000 in 2012–117,000 in 2020 [32]. Results from large-scale surveys in China indicate that there was a greater risk of overweight and obesity in the high SES group compared to the low SES group among children, adolescents, and adults, although this SES difference has narrowed [33,34]. Therefore, the findings of this study may underestimate the increase in the prevalence of overweight and obesity, and the problem of overnutrition among Chinese college students is very serious.

The prevalence of overweight and obesity among male college students was greater than that among female college students, and the magnitude and pace of the increase were greater, which was a common phenomenon in the Chinese population [3,35]. It is projected that the sex difference in the prevalence of overweight will continue to widen by 2030. First, Chinese family has a traditional perception of “son preference”, with favoring the raising of boys. Consequently, boys receive greater family financial support and food feeding since birth compared to girls, which may be associated with a greater prevalence of overweight and obesity among males [3]. This disparity may persist as children reach adulthood and enter university. Second, males are more sensitive to income inequality in terms of unfavorable health outcomes. Income inequality has increased in China over the past few decades, and when individuals experience the same change in income inequality, males are at greater risk than females for reporting poor health and a greater body mass index [36]. Third, sociocultural preferences tend to favor stronger males and “thin” females [35]. Female university students tend to overestimate their weight and restrict their diets, whereas even overweight male university students may be unaware of their weight status and may not engage in weight loss behaviors [37]. The prevalence of obesity among female college students exceeds that among males in most developing or low- and middle-income countries, but the opposite is true in Asia [7,9]. Norway [11] and Saudi Arabia [21] reported greater prevalence in males, and the opposite was true in Cameroon [12], South Korea [16], and France [19]. This sex difference may be related to the sociocultures and economic levels across countries.

Rapid economic growth was recognized as the main explanation for the rapid prevalence of overweight and obesity [1,2,9,10]. The dietary structure of Chinese adults has changed over the past decades, with a shift from a plant-based diet to an animal- and plant-based diet and an increase in the proportion of energy intake from fat [38]. Since the 21st century, the proportion of energy derived from fat in dietary intake and intake of ultra-processed foods among Chinese adults has increased, while the proportions of energy derived from protein and carbohydrates have decreased. High SES and education level groups are at risk [39,40]. This gradual Westernized dietary pattern increases the risk of overweight and obesity. Additionally, the same trend has been reported among adolescents [41], and their dietary habits may be carried into university. Developing healthy dietary and behavioral patterns during adolescence may be an important measure to curb weight gain among college students. Changes in physical activity (PA) are also important factors. The 2019 CHSSCH survey revealed that 84.8% of Chinese college students reported moderate- to vigorous-intensity PA for less than 1 hour and 75.1% of Chinese college students reported more than 2 hours of screen time [42]. Unfortunately, there is little evidence of temporal trends in PA among Chinese youth. Zang et al. [43] reported an increase in the prevalence of insufficient PA among Chinese adults from 2010 to 2018, especially among young adults (18–34 years). Interestingly, recent surveys of children and adults suggested that the trends in the prevalence of overweight and obesity among these two groups slowed after 2000 and 2010, respectively [2,44], whereas the results of this paper revealed accelerations of trends among college students. To curb the continuing increase in the prevalence of overweight and obesity among Chinese children and adolescents, China has also issued a series of policies. These include the China Dietary Nutrition Development Plan (2014–2020), the Healthy China 2030 Plan, and the 2020 Implementation Plan for the Prevention and Control of Childhood and Adolescent Obesity. These policies have yielded significant effects [2]. However, preventive measures targeting adults and college students remain limited. In 2024, the National Health Commission and 15 other departments released the Implementation Plan for the “Weight Management Year” campaign, marking a new phase in the prevention and control of obesity among adults with multisectoral collaboration and coverage across the entire life [45].

This paper revealed that rural college students in 1995 had greater risks of overweight and obesity than did urban college students, especially those who were overweight, although the detection rate was low. Presumably, rural college students who previously had to perform much physical labor before enrollment or during vacation, such as agricultural farming, may have had higher percentages of fat-free mass, and the screening criteria did not differentiate between body composition. Urban areas became high-risk areas after 2000, which was related to China's economic development and urbanization. The regional PORs showed a decreasing trend after 2000, implying that although college students in urban areas were at greater risk of overweight and obesity, the trend of prevalence in rural areas accelerated over time. These results were consistent with those of previous studies among Chinese children and adults [2,35,44]. The driving factors for urban-rural differences in China are complex. Given economic development and the convergence of urban and rural lifestyles, the environment of easier access to high-energy foods but a lack of physical activity can be extended from urban areas to rural areas. There was a greater decrease in total PA levels in rural areas than in urban areas [43]. Although China's income has steadily increased in both urban and rural areas, rural inhabitants may spend a greater proportion of their money on food than urban inhabitants do [44].

The prevalence of overweight and obesity was greater among college students aged 21–22 years than among those aged 19–20 years in recent years, and the difference in prevalence was projected to continue to widen by 2030. In Norway, the prevalence of obesity was greater and increased more rapidly among college students aged 26–35 years than among college students aged 18–25 years [11]. The prevalence of overweight/obesity in Brazilian adults aged 18–24 years has increased the most [18]. In Saudi Arabia, the prevalence of overweight increases with age [21]. It was hypothesized that unhealthy lifestyle habits developed in early adulthood increase the risk of overweight/obesity during adulthood [36]. Sedentary time and screen time are positively associated with age [42], and older college students may have a more severe overweight or obesity-causing environment. Furthermore, older college students faced greater academic pressure, spent more sedentary time meeting graduation requirements, and had the opportunity to consume more packaged and ultra-processed foods in a stress-driven environment as the economy improved.

This study has several limitations. First, the “standardized weight-for-height of 1985” criterion for designating overweight and obesity in this study was not the most commonly used criterion, but it had less influence on the analysis of temporal trends. At greater values, standardized weight-for-height was highly correlated with body fat mass, which better reflected the true status of overweight and obesity [46]. Second, the participants in this paper were college students, and many youth aged 19–22 years did not attend college. These findings should be applied with caution to the total population aged 19–22 years. Finally, data were obtained from published summary datasets, data on individuals were not available, and trends in prevalence were assessed without adjusting for factors such as biological maturity, socioeconomic status, and PA.

## Conclusions

In conclusion, the prevalence of overweight and obesity among college students aged 19–22 years in China increased significantly from 1995 to 2019, especially among male, urban, and older college students. The prevalence of overweight and obesity among rural college students will surpass that among their urban counterparts after 2027, demonstrating a reversal of the urban-rural disparity. Future policies should focus on improving diet quality, promoting physical activity, and enhancing health education among college students, as well as increasing funds and policy preferences for high-risk groups, to prevent the rapid prevalence of overweight and obesity and to improve the health and equity of young people.

## Supporting information

S1 Table Prevalence of overweight and obesity among Chinese college students aged 19–22 years by sex, region, and age, 1995–2019. Overweight was defined as an individual's weight being 110% or more of the standard weight for height, and obesity was defined as weight being 120% or more. Overweight always includes obesity.
(DOCX)
