# Supplementary material for: Trends and projections of overweight and obesity among Chinese college students from 1995 to 2019: Findings from national cross-sectional surveys
Source: PLoS One. 2026 Jan 7;21(1):e0337477. doi: 10.1371/journal.pone.0337477 (PMC12818918; doi:10.1371/journal.pone.0337477)
Supplement: S1 Table — Overweight was defined as an individual's weight being 110% or more of the standard weight for height, and obesity was defined as weight being 120% or more. Overweight always includes obesity. (DOCX) [file pone.0337477.s001.docx]

**Supplementary table 1 Prevalence of overweight and obesity among Chinese college students aged 19-22 years by sex, region, and age, 1995-2019.**

| Year | Age (yrs) | Overweight |  |  |  | Obesity |  |  |  |
| --- | --- | --- | --- | --- | --- | --- | --- | --- | --- |
|  |  | Urban boys | Urban girls | Rural boys | Rural girls | Urban boys | Urban girls | Rural boys | Rural girls |
| 1995 | 19 | 67 (2.5) | 133 (4.6) | 110 (4.0) | 140 (5.3) | 30 (1.1) | 23 (0.8) | 23 (0.8) | 18 (0.7) |
|  | 20 | 46 (1.6) | 121 (4.2) | 101 (3.6) | 117 (4.2) | 25 (0.9) | 14 (0.5) | 22 (0.8) | 13 (0.5) |
|  | 21 | 55 (2.0) | 109 (3.8) | 119 (4.2) | 115 (4.2) | 20 (0.7) | 12 (0.4) | 20 (0.7) | 11 (0.4) |
|  | 22 | 55 (2.1) | 113 (4.1) | 144 (5.0) | 103 (36.3) | 21 (0.8) | 20 (0.7) | 26 (0.9) | 9 (0.3) |
| 2000 | 19 | 518 (15.2) | 235 (6.6) | 281 (8.4) | 192 (5.9) | 156 (4.6) | 72 (2) | 96 (2.9) | 37 (1.1) |
|  | 20 | 488 (14.9) | 218 (6.4) | 232 (6.8) | 169 (5.4) | 163 (5.0) | 52 (1.5) | 72 (2.1) | 26 (0.8) |
|  | 21 | 526 (16.0) | 191 (6.2) | 275 (7.7) | 175 (5.5) | 195 (5.9) | 46 (1.5) | 87 (2.5) | 43 (1.4) |
|  | 22 | 448 (15.9) | 147 (5.5) | 333 (10.6) | 173 (6.5) | 150 (5.3) | 33 (1.2) | 72 (2.3) | 30 (1.1) |
| 2005 | 19 | 656 (17.4) | 300 (7.6) | 358 (9.8) | 219 (5.9) | 256 (6.8) | 88 (2.2) | 119 (3.3) | 43 (1.2) |
|  | 20 | 692 (18.5) | 274 (7.0) | 371 (10.2) | 234 (6.6) | 261 (7.0) | 77 (2.0) | 123 (3.4) | 53 (1.5) |
|  | 21 | 687 (18.4) | 275 (7.3) | 399 (11.5) | 173 (5.2) | 283 (7.6) | 67 (1.8) | 105 (3.0) | 36 (1.1) |
|  | 22 | 678 (20.4) | 188 (5.5) | 399 (12.5) | 133 (4.3) | 247 (7.4) | 51 (1.5) | 131 (4.1) | 29 (0.9) |
| 2010 | 19 | 679 (22.9) | 251 (8.4) | 471 (15.7) | 185 (6.2) | 283 (9.6) | 71 (2.4) | 173 (5.8) | 28 (0.9) |
|  | 20 | 651 (21.9) | 200 (6.7) | 442 (14.8) | 192 (6.4) | 270 (9.1) | 58 (2.0) | 172 (5.7) | 62 (2.1) |
|  | 21 | 690 (23.3) | 222 (7.4) | 508 (17.0) | 166 (5.5) | 246 (8.3) | 61 (2.0) | 156 (5.2) | 36 (1.2) |
|  | 22 | 722 (24.8) | 173 (5.9) | 525 (17.9) | 174 (5.9) | 295 (10.1) | 47 (1.6) | 190 (6.5) | 42 (1.4) |
| 2014 | 19 | 788 (26.6) | 303 (10.1) | 569 (19.1) | 261 (8.7) | 324 (10.9) | 100 (3.3) | 211 (7.1) | 80 (2.7) |
|  | 20 | 818 (27.6) | 319 (10.7) | 581 (19.5) | 265 (8.8) | 372 (12.6) | 98 (3.3) | 229 (7.7) | 72 (2.4) |
|  | 21 | 908 (30.4) | 272 (9.1) | 619 (20.8) | 261 (8.7) | 378 (12.7) | 99 (3.3) | 233 (7.8) | 77 (2.6) |
|  | 22 | 894 (31.3) | 269 (9.3) | 729 (24.5) | 234 (7.8) | 356 (12.5) | 96 (3.3) | 280 (9.4) | 50 (1.7) |
| 2019 | 19 | 980 (35.3) | 425 (15.0) | 720 (26.8) | 372 (13.5) | 526 (19.0) | 189 (6.7) | 346 (12.9) | 159 (5.8) |
|  | 20 | 1019 (35.6) | 434 (15.2) | 789 (28.3) | 361 (12.6) | 519 (18.1) | 192 (6.7) | 383 (13.7) | 144 (5.0) |
|  | 21 | 1052 (38.0) | 367 (13.2) | 860 (30.9) | 374 (13.2) | 572 (20.6) | 165 (5.9) | 410 (14.7) | 147 (5.2) |
|  | 22 | 965 (37.9) | 389 (14.9) | 793 (32.5) | 312 (12.6) | 500 (19.6) | 170 (6.2) | 403 (16.5) | 130 (5.3) |

Overweight was defined as an individual's weight being 110% or more of the standard weight for height, and obesity was defined as weight being 120% or more. Overweight always includes obesity.
